# PD-1 blockade delays tumor growth by inhibiting an intrinsic SHP2/Ras/MAPK signalling in thyroid cancer cells

**DOI:** 10.1186/s13046-020-01818-1

**Published:** 2021-01-07

**Authors:** Federica Liotti, Narender Kumar, Nella Prevete, Maria Marotta, Daniela Sorriento, Caterina Ieranò, Andrea Ronchi, Federica Zito Marino, Sonia Moretti, Renato Colella, Efiso Puxeddu, Simona Paladino, Yoshihito Kano, Michael Ohh, Stefania Scala, Rosa Marina Melillo

**Affiliations:** 1 Institute of Experimental Endocrinology and Oncology (IEOS), CNR, Naples, Italy; 2 Department of Translational Medical Sciences, University of Naples Federico II, Naples, Italy; 3 Department of Molecular Medicine and Medical Biotechnology, University of Naples Federico II, Via S. Pansini 5, 80131 Naples, Italy; 4 Department of Advanced Biomedical Sciences, University of Naples Federico II, Naples, Italy; 5 Functional Genomics, Istituto Nazionale Tumouri “Fondazione G. Pascale”, IRCCS, Naples, Italy; 6 Department of Mental and Physical Health and Preventive Medicine, University of Campania “Luigi Vanvitelli”, Naples, Italy; 7 Department of Medicine, University of Perugia, Perugia, Italy; 8 Department of Experimental Medicine, University of Perugia, Perugia, Italy; 9 Department of Clinical Oncology, Graduate School of Medical and Dental Sciences, Tokyo Medical and Dental University, Tokyo, Japan; 10 Department of Laboratory Medicine and Pathobiology, University of Toronto, Toronto, Canada; 11 Department of Biochemistry Faculty of Medicine, University of Toronto, Toronto, Canada

**Keywords:** Thyroid Cancer, Programmed cell death-1, SHP2 phosphatase, Ras, MAPK signalling

## Abstract

**Background:**

The programmed cell death-1 (PD-1) receptor and its ligands PD-L1 and PD-L2 are immune checkpoints that suppress anti-cancer immunity. Typically, cancer cells express the PD-Ls that bind PD-1 on immune cells, inhibiting their activity. Recently, PD-1 expression has also been found in cancer cells. Here, we analysed expression and functions of PD-1 in thyroid cancer (TC).

**Methods:**

PD-1 expression was evaluated by immunohistochemistry on human TC samples and by RT-PCR, western blot and FACS on TC cell lines. Proliferation and migration of TC cells in culture were assessed by BrdU incorporation and Boyden chamber assays. Biochemical studies were performed by western blot, immunoprecipitation, pull-down and phosphatase assays. TC cell tumorigenicity was assessed by xenotransplants in nude mice.

**Results:**

Human TC specimens (47%), but not normal thyroids, displayed PD-1 expression in epithelial cells, which significantly correlated with tumour stage and lymph-node metastasis. PD-1 was also constitutively expressed on TC cell lines. PD-1 overexpression/stimulation promoted TC cell proliferation and migration. Accordingly, PD-1 genetic/pharmacologic inhibition caused the opposite effects. Mechanistically, PD-1 recruited the SHP2 phosphatase to the plasma membrane and potentiated its phosphatase activity. SHP2 enhanced Ras activation by dephosphorylating its inhibitory tyrosine 32, thus triggering the MAPK cascade. SHP2, BRAF and MEK were necessary for PD-1-mediated biologic functions. PD-1 inhibition decreased, while PD-1 enforced expression facilitated, TC cell xenograft growth in mice by affecting tumour cell proliferation.

**Conclusions:**

PD-1 circuit blockade in TC, besides restoring anti-cancer immunity, could also directly impair TC cell growth by inhibiting the SHP2/Ras/MAPK signalling pathway.

## Background

Immunotherapy represents the major breakthrough of the last years in the therapy of several cancer types [1]. The programmed cell death-ligand 1 and 2 (PD-L1, PD-L2) are immune checkpoints (IC) important for delivering inhibitory signals to immune cells expressing their receptor programmed cell death-1 (PD-1) [1, 2]. This circuit is critical in regulating immune tolerance in various physiologic and pathologic contexts [1]. Cancer cells suppress anti-cancer immune response exploiting the PD-1 circuit [3]. Typically, PD-Ls are expressed by cancer cells, while PD-1 is expressed by immune cells with anti-cancer potential (i.e., T cells, macrophages or natural killer cells) [3]. The inhibition of this circuit through immune checkpoint inhibitors (ICI) - neutralizing antibodies against PD-1, PD-L1 or PD-L2 - restores the anti-cancer immune response and displays therapeutic activity in various cancer types [4].

Recently, various tumour types have been found to express also intrinsic PD-1 (i.e., melanoma, hepatocarcinoma, lung carcinoma and T-cell lymphomas) [5–8]. PD-1 intrinsic signalling promoted tumour growth in melanoma and hepatocarcinoma through a mammalian target of rapamycin (mTOR)/ribosomal protein S6 Kinase (S6K1) pathway [5, 6]. By contrast, in non-small cell lung cancer (NSCLC) and in T-cell lymphomas, PD-1 behaved as a tumour suppressor [7, 8]. These data indicate that PD-1 could exert context-related tumour-intrinsic functions other than the suppression of immune response, and suggest the need of wider studies on ICI effects on the entire tumour context.

Thyroid carcinoma (TC) is the most frequent endocrine malignancy. Follicular cell-derived TC includes different histotypes ranging from well differentiated (WDTC) to poorly differentiated (PDTC) and undifferentiated/anaplastic (ATC) carcinomas. WDTCs include papillary histotype (PTC), representing the majority of these tumours, and follicular histotype (FTC). WDTCs show an indolent behaviour and are mainly cured by surgery and ^131^I radioiodine (RAI) therapy; only a small percentage of them exhibits recurrence, metastasis and resistance to RAI over time. By contrast, aggressive forms of TC (PDTC and ATC) represent a clinic challenge displaying a remarkable chemo- and radio-resistant phenotype from the beginning [9, 10]. Interestingly, aggressive forms of TC exhibit increased immune checkpoint expression and inefficient immune infiltrate [9, 11–14], features that are being evaluated for the treatment of the disease [9, 14, 15].

Here, we analysed the PD-1/PD-Ls circuit in TC showing that: i) TC cell lines and TC human samples express, besides PD-Ls, as already demonstrated [16–18], also PD-1 in epithelial cells, whose levels correlated with tumour aggressiveness; ii) intrinsic PD-1 sustains proliferation and migration of TC cells through a SHP2/Ras/MAPK signalling cascade; iii) PD-1 overexpression promotes, while PD-1 blockade inhibits, ATC xenograft growth by affecting cancer cell proliferation.

Thus, TCs express an intrinsic pro-tumorigenic PD-1 circuit. In TC context, the oncogenic role of PD-1 is dependent on the activation of the Ras/MAPK cascade. PD-1 blockade may represent a rational therapeutic choice in aggressive forms of TC for both immune response reconstitution and direct anti-tumour effects.

## Materials and methods

### Reagents

pCMV3 and pCMV3 PD-1 plasmids were from Sinobiological (Wayne, PA, USA), pCEFL and pCEFL AU5-tagged Ras (V12) plasmids were a kind gift of J.S. Gutkind [19]. PD-1 was cloned in pFLAG 5A (Invitrogen, Carlsbad, CA, USA). Soluble PD-L1 (sPD-L1) was from R&D systems (Minneapolis, MN, USA), Nivolumab was kindly provided by S. Scala. Anti-Ras antibody for immunoprecipitation (clone MA1012) was from Invitrogen. Anti-phospho Y32, anti-phospho Y64 Ras antibodies and Y32 and Y64 peptides, used to saturate aspecific binding of each antibody, were provided by M. Ohh. SHP099, Vemurafenib, and Selumetinib were from Selleckchem (Houston, TX, USA). IgG_4_ control antibodies were from Invitrogen.

### Cell culture and transfection

Human thyroid cancer cell lines BcPAP, TPC-1, 8505c, CAL62, SW1736, FRO, BHT101, HTH7 and OCUT1 were obtained and maintained as previously described [20]. The normal thyroid cells H-6040, isolated from normal human thyroid tissue and cultured in Human Epithelial Cell Medium with the addition of Insulin-Transferrin-Selenium, EGF, Hydrocortisone, L-Glutamine, antibiotic-antimycotic solution, Epithelial Cell supplement, and FBS were purchased from Cell Biologics (Chicago, IL, USA). H-6040 cells were used at passages between 3 and 6.

Transient transfections of TC cells were performed using polyethylenimine according to manufacturer’s instructions (Merck, Darmstadt, Germany). Cells were harvested 48 h after transfection. Electroporation was used (Neon® Transfection System for Electroporation, Life Technologies, Carlsbad, CA, USA) to obtain stably transfected cells [21].

For RNA interference, we used SMART pools of siRNA from Dharmacon (Lafayette, CO, USA) targeting PD-1 or SHP2. Transfection was carried out by using 100 nM of SMARTpool and 6 μl of DharmaFECT (Dharmacon) for 48 h [22].

### Cytofluorimetric analysis

Cells were incubated (30 min at 4 °C) with specific or isotype control antibodies. Cells were analysed with a FACS Calibur cytofluorimeter using CellQuest software (BD Biosciences, Mississauga, ON, Canada). 10^4^ events for each sample were acquired [22]. Anti-PD-1 and anti-PD-L1 antibodies were from ebioscience (Thermo Fisher, Waltham, MA, USA), anti-PD-L2 from Miltenyi Biotec (Bergisch Gladbach, Germany).

### Immunohistochemistry

Thyroid carcinomas were selected from the Pathology Unit of the University of Perugia upon informed consent; the protocol for the study was approved by the institutional committee of University of Perugia. Thyroid tissues were formalin fixed and paraffin embedded (FFPE). Sections of 4 μm were obtained. BOND-III fully automated immunohistochemistry stainer (Leica Biosystems, Wetzlar, Germany) carried out the immunostaining, using heat-induced antigen retrieval at pH 9.0 for 20 min, followed by primary antibody (PD-1, clone EH33; dilution 1:200) (Cell Signaling, Beverly, MA, USA) incubation for 15 min. Finally, the ready to use Bond™ Polymer Refine Detection System allowed the detection of antigen-antibody reaction [11]. Immunohistochemical (IHC) score was calculated by combining the staining intensity with the percentage of immuno-reactive epithelial cells. Staining intensity was rated on a scale of 0–3 (0, negative; 1, weak; 2, moderate; and 3, strong). Each tumor was then scored for the percentage of immuno-reactive cells. The IHC score was then assigned to each tumor by multiplying the percentage of positive epithelial cells for the staining intensity. The IHC score ranged from 0 to 300. We used a cut-off of 5% to determine the positivity of immunohistochemistry, consistently with the cut-off chosen to evaluate PD-1 in other cancer systems [6, 23, 24].

### S-phase entry

S-phase entry was evaluated by Bromodeoxyuridine (BrdU) incorporation. Cells were serum-deprived and treated with stimuli for 24 h. BrdU was added at a concentration of 10 μM for the last 1 h. BrdU-positive cells were revealed with Texas Red conjugated secondary Abs (Jackson Laboratories, West Grove, PA, USA). Fluorescence was detected by FACS analysis [25].

### Migration assays

Chemotaxis was evaluated using a Boyden chamber. We used a 48-well microchemotaxis chamber (NeuroProbe, Gaithersburg, MD, USA) and 8-μm-pore polycarbonate membranes (Nucleopore, Pleasanton, CA, USA) coated with 10 μg/ml fibronectin (Merck) as described elsewhere [22].

### Protein studies

Protein extraction and immunoblotting experiments were performed according to standard procedures [26]. Antibodies to PD-1, phospho-PD-1, phospho-BRAF, phospho-MEK1/2, phospho-MAPK (p44/p42), Ras, phospho-SHP2, SHP2, and GRB2 for Western blot analysis were obtained from Cell Signaling Technology (Danvers, MA, USA). Monoclonal anti-tubulin antibody was from Sigma Aldrich. Secondary anti-mouse and anti-rabbit antibodies were coupled to horseradish peroxidase (Biorad, Hercules, CA, USA).

Cell lysates were subjected to immunoprecipitation with different antibodies or subjected to pull-down binding assays with purified recombinant proteins immobilized on agarose beads. The glutathione-S-transferases (GST) fusion proteins were expressed in *Escherichia coli* and purified with glutathione-conjugated agarose beads (Merck) by standard procedures. The protein complexes were eluted and resolved by sodium dodecyl sulphate-polyacrylamide gel electrophoresis (SDS-PAGE). Immunoblotting with specific antibodies and enhanced chemiluminescence (ECL; Thermo Fisher) were employed for immune-detection of proteins in complexes [27].

Cell fractionation experiments were performed using the Subcellular Protein Fractionation Kit for Cultured Cells according to manufacturer’s instructions (Thermo Fisher). Membrane fraction’s protein content was normalized by using anti-transferrin receptor antibody (Invitrogen).

### Immunofluorescence

Cells, grown on coverslips, were washed with phosphate-buffered saline (PBS), fixed with 4% paraformaldehyde (PFA) and quenched with 50 mM NH_4_Cl. Then, cells were permeabilized with 0.2% Triton X-100 for 5 min and blocked for 30 min in PBS containing 5% FBS and 0.5% bovine serum albumin (BSA). Primary antibodies were detected with Alexa Fluor546-conjugated secondary antibodies (Abcam, Cambrige, UK). Images were acquired using a laser scanning confocal microscope (LSM 510; Carl Zeiss MicroImaging, Inc., Oberkochen, Germania.) equipped with a planapo 63X oil-immersion (NA 1.4) objective lens by using the appropriate laser lines and setting the confocal pinhole to one Airy unit. Z-slices from the top to the bottom of the cell by using the same setting (laser power, detector gain) were collected as previously described [28].

### SHP2 activity assay

SHP2 phosphatase activity was determined using the human/mouse/rat active DuoSet IC kit (R&D Systems). Briefly, cellular SHP2 bound to anti-SHP2 antibody conjugated to agarose beads was exposed to synthetic phosphopeptide substrate, which is only dephosphorylated by active SHP2. The amount of free phosphate generated in the supernatant was determined, as absorbance at 620 nm, by a sensitive dye-binding assay using malachite green and molybdic acid and represents a direct measurement of SHP2 activity in the cellular system [29].

### Tumorigenicity in immunocompromised mice

Each group of 8 mice (6-week-old CD1 nu/nu females) was inoculated subcutaneously with 8505c parental cells, 8505c transfected with pCMV3 or pCMV3 PD-1 cells (1x10^7^cells/mouse, two clones) [25]. Nivolumab (anti-PD-1) or control IgG_4_ were intraperitoneally (i.p.) administered at 30 mg/kg twice per week. The experimental protocol for animal studies was approved by the Ministero Italiano della Salute (No. 317/2019-PR). For xenograft histological analysis, anti-Ki-67 was from Biocare Medical (Pacheco, CA, USA), anti-CD31, anti-cleaved caspase 3 were from R&D Systems.

### Statistical analysis

The results are expressed as the mean ± SD of at least 3 experiments. Values from groups were compared using the paired Student *t* test or Duncan test. The association between PD-1 expression and clinic-pathologic parameters in immunohistochemistry experiments was conducted using χ^2^. *P* value < 0.05 was considered statistically significant.

## Results

### PD-1 receptor and its ligands are expressed in thyroid carcinoma cells

**Fig. 1:**
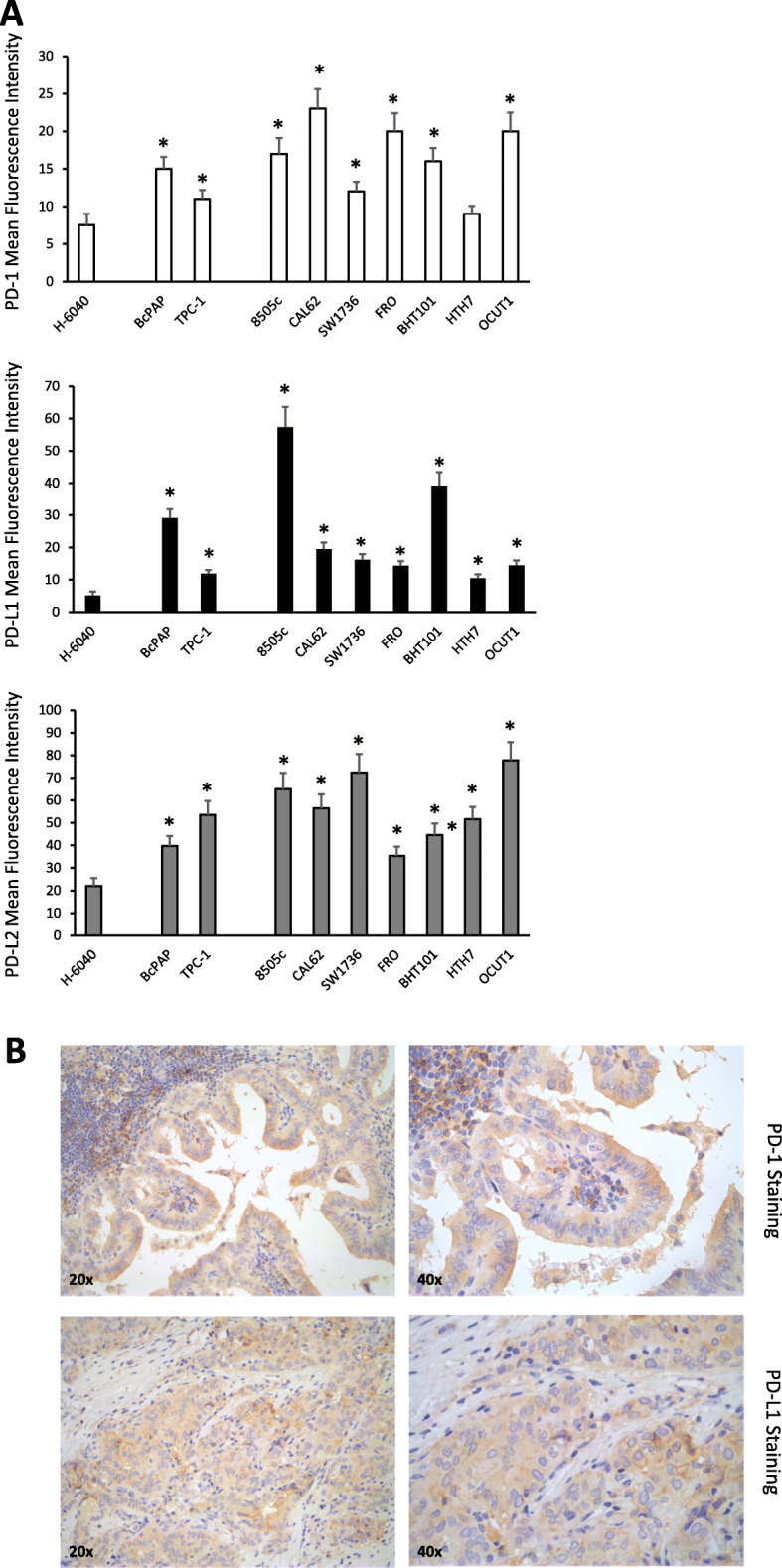
Immune checkpoint expression in thyroid cancer (TC) cells and human TC tissue samples. **a.** Mean Fluorescence Intensity for PD-1, PD-L1 and PD-L2 measured by flow cytometric analysis on H-6040 normal thyroid epithelial cells, PTC-derived cell lines (BcPAP and TPC-1), and ATC-derived cell lines (8505c, CAL62, SW1736, FRO, BHT101, HTH7, OCUT1). Data are presented as mean ± SD. * *P* < 0.05 compared to the H-6040 cells. **b.** Immunohistochemical staining of representative PTC samples with anti-PD-1 or anti-PD-L1 antibodies (20x and 40x). In the upper panels, PD-1 immunoreactivity in both thyroid epithelial cells and in infiltrating lymphocytes can be detected. Lower panels show PD-L1 immunoreactivity in a representative PTC sample

We evaluated the expression levels of PD-1, PD-L1 and PD-L2 in a panel of human TC cell lines derived from PTC (BcPAP, TPC-1) or ATC (8505c, CAL62, SW1736, FRO, BHT101, HTH7, OCUT1) compared to a primary human thyroid cell culture (H-6040). Cytofluorimetric analysis demonstrated that all the cell lines expressed PD-1 on the plasma membrane, though to a lesser extent than PD-Ls, and that PD-1 protein levels were higher in cancer compared to normal thyroid cells (Fig. 1a). Similar data were also obtained through western blot analysis as shown in Supplementary Figure 1**A**. PD-1, PD-L1 and PD-L2 mRNA levels were comparable between normal and cancerous thyroid cells, suggesting that post-translational mechanisms could be responsible for the protein increase observed in cancer cells (Suppl. Fig. 1b).

Immunohistochemical (IHC) staining of whole sections from 34 PTC surgical samples with anti-PD-1 antibodies showed that PD-1 is expressed in TC cells (Fig. 1b), but not in normal thyroid epithelial cells (**not shown**). Figure 1b shows a representative PTC case with PD-1 immunoreactivity, cytosolic and/or localized at the plasma membrane, in thyroid cancer epithelial cells (20x and 40x) (Fig. 1b). Infiltrating lymphocytes showed a stronger PD-1 staining compared to cancer cells. PD-1 expression was detectable in epithelial cancerous cells of 47% of tumour samples (Table 1). By analysing clinic-pathologic features of the PTC samples, we found that tumour stage and lymph-nodal metastasis significantly correlated with PD-1 staining (Table 1) in our casistic.

**Table 1:** Relation between PD-1 epithelial TC cell expression and clinic-pathological features. * *P* < 0.05 among groups

	Epithelial cell PD-1 staining		***P***
	Negative/low	Positive	
**BRAF (*n*** **= 34)**			
Not mutated	2	2	0.9
Mutated	16	14	
**Thyroiditis (*n*** **= 33)**			
No	12	11	0.9
Yes	5	5	
**TNM (*n*** **= 34)**			
T1	11	9	
T2	4	3	0.77
T3	3	4	
N0	17	6	
N1	1	10	0.04*
M0	11	15	
M1	7	1	0.9
**Progression free survival (*n*** **= 28)**			
No	3	4	
Yes	12	9	0.51
**Stage (*n*** **= 29)**			
1	12	6	
2	0	0	0.04*
3	1	5	
4	2	3	
**Infiltrative phenotype (*n*** **= 34)**			
No	15	13	
Yes	3	3	0.87

Several reports already described the expression of PD-L1 in TC cells of human samples and demonstrated that its expression is associated with aggressiveness, suggesting that PD-L1 could represent a useful prognostic marker for TC patients [16–18]. We also confirmed in our casistic the expression of the PD-L1 in thyroid cancer epithelial cells (Fig. 1b).

Altogether, our data on TC samples, on TC cell lines and the data available from the literature indicate that TC cells can express PD-1 together with its ligands. Our results also indicate that PD-1 expression correlates with tumour malignancy.

### PD-1 promotes thyroid carcinoma cell proliferation and motility

We selected 8505c and TPC-1 cells - derived from a human ATC and PTC, respectively - to analyse the biologic effects of PD-1 enforced expression or of PD-1 stimulation by soluble PD-L1 (sPD-L1–1 μg/ml). Levels of PD-1 expression upon transient transfection are shown in Suppl. Fig. 2a. The endogenous PD-1 protein expression levels, already shown in TC wild-type cells (Suppl. Fig. 1A**)**, are not evident in the empty-vector transfected cells due to the short time of exposure (Suppl. Fig. 2A**)**.

**Fig. 2:**
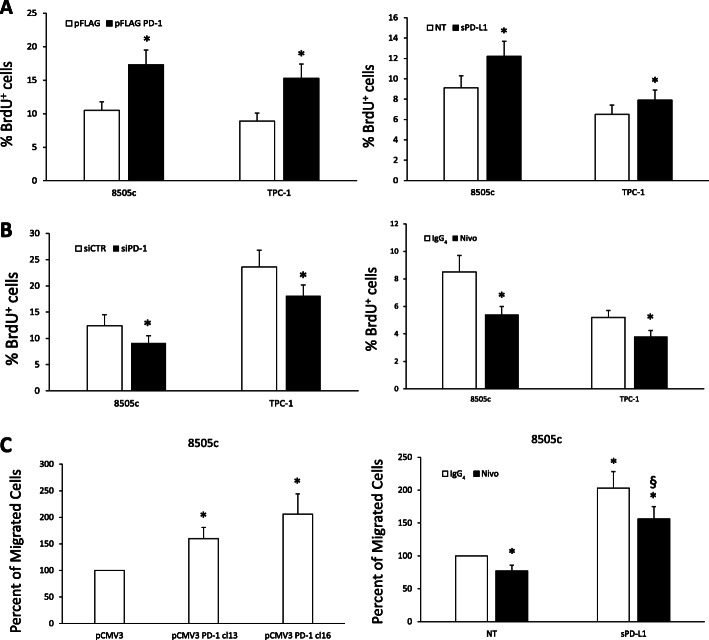
Functional activity of intrinsic PD-1 in TC cells. **a.** DNA synthesis of 8505c and TPC-1 cells transiently transfected with pFLAG PD-1 or the relative empty vector (pFLAG), or treated or not with soluble PD-L1 (sPD-L1–1 μg/ml) assessed by BrdU incorporation. Data are presented as mean ± SD of 5 independent experiments. **b.** DNA synthesis of 8505c and TPC-1 cells treated with siRNA targeting PD-1 (siPD-1 – 100 nM) or the relative control (siCTR – 100 nM) for 48 h or treated with Nivolumab (Nivo - 10 μg/ml) or control IgG_4_ (10 μg/ml) for 24 h assessed by BrdU incorporation. Data are presented as mean ± SD of 5 independent experiments. **c.** Percent of migrated cells over control (empty vector transfected – pCMV3) of stably transfected 8505c PD-1 cells versus 10% FBS, or of 8505c cells treated with Nivolumab (Nivo - 10 μg/ml) or control IgG_4_ (10 μg/ml) toward sPD-L1 (1 μg/ml) or medium alone (NT). Data are presented as mean ± SD of 5 independent experiments. * *P* < 0.05 compared to the relative untreated cells. § *P* < 0.05 compared to sPD-L1 alone

We demonstrated that transient PD-1 overexpression (pFLAG PD-1 compared to pFLAG) or PD-1 activation (sPD-L1 vs untreated - NT) significantly increased DNA synthesis, as assessed by BrdU incorporation (Fig. 2a) in both TC cell lines. Accordingly, cell cycle analysis showed an increased percentage of cells in S and G2/M phases in PD-1-transfected compared to empty vector-transfected TC cells (Suppl. Fig. 2B). No effects of PD-1 overexpression/activation were observed on cell viability (Suppl. Fig. 2C).

In order to confirm these observations, we evaluated the effects of PD-1 inhibition on the same cellular functions. To this aim, PD-1 expression was inhibited by siRNA or Nivolumab (anti-PD-1 moAb) in TPC-1 and 8505c cells. Both siRNAs targeting PD-1 (siPD-1 vs siCTR - 100 nM; Suppl. Fig. 2D) and Nivolumab (Nivo - 10 μg/ml) were able to significantly inhibit BrdU incorporation (Fig. 2b) and cell cycle progression (Suppl. Fig. 2E) of TC cells in comparison to the relative controls, without affecting cell viability (Suppl. Fig. 2F).

To assess the role of endogenous PD-1 ligands in TC cell proliferation, we treated 8505c cells with blocking anti-PD-L1 or anti-PD-L2 moAb (10 μg/ml) or transiently transfected them with PD-L1 or PD-L2 expressing vectors. PD-L1 or PD-L2 overexpression increased, while anti-PD-L1 or anti-PD-L2 antibodies inhibited, BrdU incorporation in 8505c cells (Suppl. Fig. 2G). No effects of PD-L1 or PD-L2 were observed on TC cell viability (**not shown**).

Since PD-1 expression levels in human TC samples correlated with lymph-nodal metastasis, we asked whether PD-1 could also stimulate the motility of TC cells. To this aim, we performed migration assays on 8505c cells stably overexpressing or not PD-1 [pCMV3 PD-1 cl13 and cl16 compared to pCMV3 empty vector-transfected cells (Suppl. Fig. 3A)] or on parental 8505c cells treated or not with sPD-L1 (1 μg/ml) in the presence or absence of Nivolumab (Nivo - 10 μg/ml) (Fig. 2c). PD-1 overexpressing TC cells showed an increased constitutive migratory potential compared to control cells. Consistently, sPD-L1 induced, and Nivolumab inhibited, both basal and sPD-L1-induced migration (Fig. 2c).

These data indicate that PD-1 intrinsic circuit sustains TC cell proliferation and migration.

### PD-1 activates the Ras/MAPK signalling cascade in thyroid carcinoma cells

**Fig. 3:**
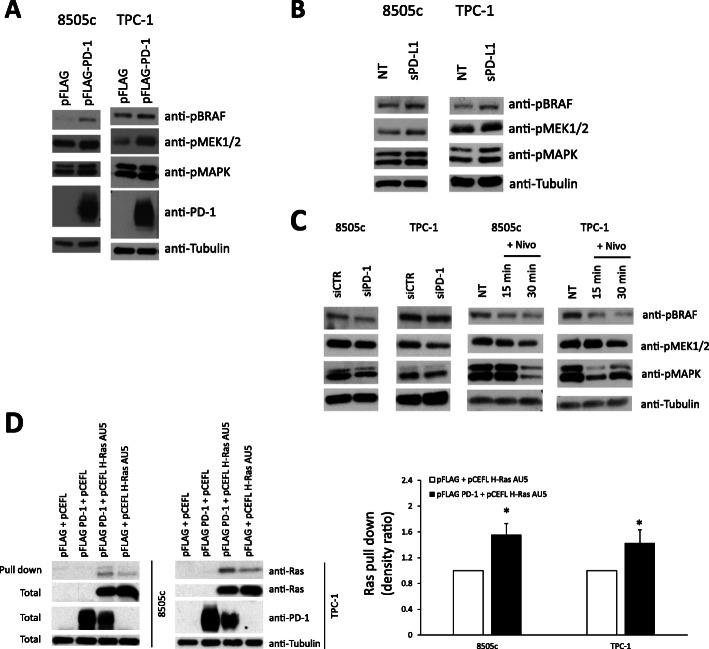
Signalling pathways downstream PD-1 activation/overexpression. **a.** Activation of BRAF, MEK1/2 and MAPK (p44/p42) in 8505c and TPC-1 cells, transfected with pFLAG PD-1 or the relative empty vector (pFLAG), assessed by western blot for their phosphorylated forms. A representative experiment is shown. **b.** Activation of BRAF, MEK1/2 and MAPK in 8505c and TPC-1 cells, treated or not with sPD-L1 (1 μg/ml - 30 min), assessed by western blot for their phosphorylated forms. A representative experiment is shown. **c.** Activation of BRAF, MEK1/2 and MAPK in 8505c and TPC-1 cells, treated with siPD1 or siCTR (100 nM - 48 h) or with Nivolumab or IgG_4_ (10 μg/ml – 15 and 30 min), assessed by western blot for their phosphorylated forms. A representative experiment is shown. **d.** Pull-down assay with the GST-RAF1-Ras Binding Domain (RBD) of 8505c and TPC-1 cells transiently transfected with pFLAG + pCEFL, pFLAG PD-1 + pCEFL, pFLAG + pCEFL H-Ras AU5, or pFLAG PD-1 + pCEFL H-Ras AU5. A representative pull-down is shown, together with the mean densitometric analysis ± SD of 5 independent assays. * *P* < 0.05 compared to the relative control

We then asked which signalling pathway was stimulated upon PD-1 overexpression/activation. To this aim, we used specific phospho-antibodies against various signalling proteins. We found that BRAF, MEK and MAPK (p44/p42) are activated, as demonstrated by the increased levels of their phosphorylated forms, upon PD-1 transient transfection (Fig. 3a), PD-1 stable transfection (Suppl. Fig. 3B), and sPD-L1 treatment (Fig. 3b) in both 8505c and TPC-1 cells. No significant activation of other signalling proteins was detected (Suppl. Fig. 3C). To confirm these observations, BRAF, MEK1/2 and MAPK activation levels were evaluated upon PD-1 blockade by siPD-1 or Nivolumab treatment. Consistently, both siPD-1 (100 nM) and Nivolumab (10 μg/ml - 15 and 30 min) reduced the levels of phosphorylated BRAF, MEK1/2 and MAPK compared to the relative controls (Fig. 3c) in TC cells.

Since the BRAF/MEK/MAPK signalling is potentiated by PD-1 in TC cells, and Ras GTPase is the main upstream activator of this cascade [30], we asked whether PD-1 could activate Ras. To this end, we used a pull-down assay with the GST-RAF1-Ras binding domain (RBD), which specifically binds the GTP-loaded active form of Ras. 8505c and TPC-1 cells were transiently transfected with empty vector (pFLAG) or PD-1 (pFLAG PD-1) in combination with pCEFL H-Ras AU5 or the relative empty vector (pCEFL). PD-1 enforced expression increased Ras activation, as assessed by Ras pull-down, in comparison to control (Fig. 3d**)**, suggesting that PD-1 potentiates Ras activation in TC cells.

### PD-1 recruits and activates the SHP2 phosphatase in thyroid carcinoma cells

In immune cells, PD-1 signalling requires the tyrosine phosphatase SHP2 (PTPN11) [29]. Upon phosphorylation of tyrosine residues in its cytosolic domain, PD-1 binds to the SH2 domains of SHP2 that, in turn, dephosphorylates signalling components of the immune receptors, thus down-regulating the immune responses [31]. In cancer cells, SHP2 acts as a signalling molecule downstream receptor tyrosine kinases (RTKs), displaying oncogenic activity [32]. In particular, SHP2 can contribute to Ras activation either by recruiting the GRB2/SOS complex to the plasma membrane [33] or through its phosphatase activity on Ras inhibitory tyrosine residues [33, 34].

**Fig. 4:**
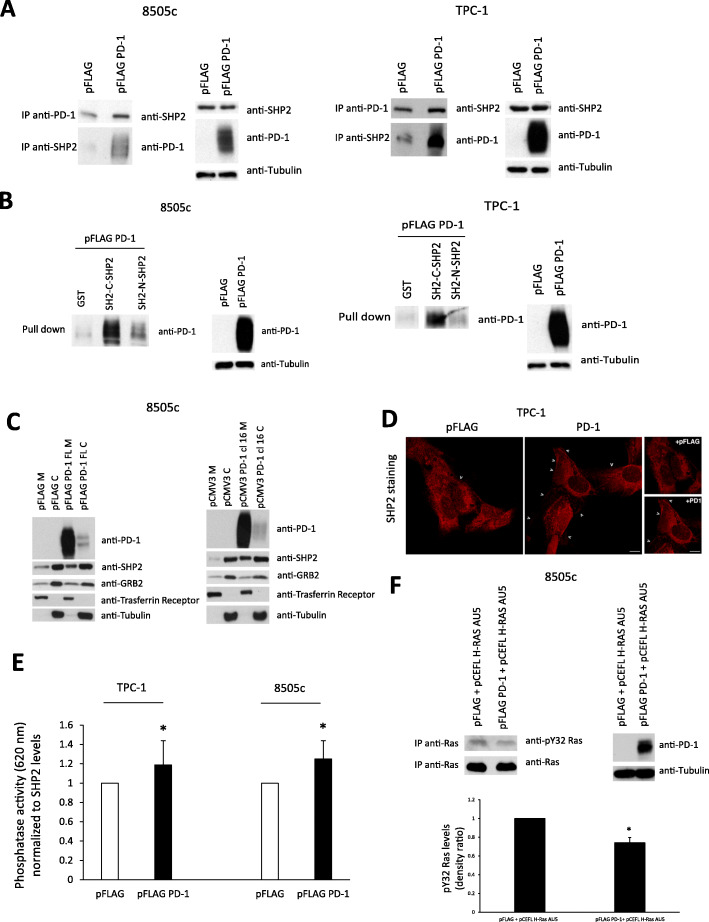
Effects of intrinsic PD-1 on SHP2 localization and functions. **a.** Total cell protein extracts from 8505c and TPC-1 cells transiently transfected with pFLAG PD-1 or the empty vector (pFLAG) were subjected to immunoprecipitation followed by western blotting with the indicated antibodies. A representative experiment is shown. **b.** Total protein extracts from 8505c and TPC-1 cells transiently transfected with pFLAG-PD-1 were subjected to an in vitro pull-down assay using the indicated recombinant proteins. Bound proteins were immunoblotted with antibody against PD-1. A representative experiment is shown. **c.** 8505c cells transiently transfected with PD-1 (pFLAG PD-1) or stably overexpressing PD-1 (pCMV3 PD-1 cl16) and the relative control cells were harvested and subjected to cell protein fractionation. Membrane (M) and cytoplasmic (C) protein fractions were immunoblotted with the indicated antibodies. Transferrin receptor or tubulin levels were used as normalization of membrane and cytosolic fractions, respectively. A representative experiment is shown. **d.** Immunofluorescence microscopy of TPC-1 cells, transiently transfected with pFLAG PD-1 or the empty vector, stained with an antibody specific for SHP2. A representative experiment is shown. Arrows indicate the surface signal of SHP2. Bars, 5 μm. **e.** SHP2 phosphatase activity assay on TPC-1 and 8505c cells transiently transfected with PD-1 (pFLAG PD-1) and the relative control (pFLAG), assessed by using a specific SHP2 phosphorylated substrate in the presence of the Malachite Green tracer, a colorimetric method (absorbance at 620 nm) for the detection of free inorganic phosphate. The SHP2 phosphatase activity was normalized for SHP2 content as assessed by western blot. Data are presented as mean ± SD of 3 independent experiments. **f.** Total cell protein extracts from 8505c cells transiently transfected with pCEFL H-Ras AU5 + pFLAG PD-1 or empty vector (pFLAG) were subjected to immunoprecipitation followed by western blotting with the indicated antibodies. A representative experiment is shown, together with the mean densitometric analysis ± SD of 5 independent assays. * *P* < 0.05 compared to the relative control

We first asked whether PD-1 could physically interact with SHP2 in TC cells. Reciprocal co-immunoprecipitation experiments showed that endogenous and exogenously expressed PD-1 bind SHP2 in 8505c and TPC-1 cells (Fig. 4a). Moreover, pull-down assays with N- or C-terminal SH2 domain of SHP2 demonstrated that SHP2 can bind PD-1 mainly through SHP2 C-terminal SH2 domain (Fig. 4b). In support of these observations, we found that both endogenous and exogenous PD-1 are tyrosine phosphorylated in TC cells (Suppl. Fig. 4A), condition necessary to allow the SH2 domains of SHP2 to bind PD-1 [33].

Cell fractionation of 8505c cells transiently or stably transfected with PD-1 was used to demonstrate that PD-1 binding to SHP2 enforced the membrane localization of SHP2. Subcellular fractions of membranes (M) or cytosol (C) were obtained from PD-1 overexpressing and from control cells (pFLAG-PD-1 vs pFLAG or pCMV3 PD-1 cl 16 vs pCMV3). Enrichment of SHP2 levels in the membrane fractions was observed in PD-1 overexpressing cells compared to empty-vector transfected cells. Normalizations of each extract were obtained by using antibodies to transferrin receptor for membrane fraction and anti-tubulin for cytosolic extract (Fig. 4c). In agreement with these observations, immunofluorescence (IF) assay of PD-1 overexpressing TC cells showed a significant increase of SHP2 staining at the plasma membrane in cells overexpressing PD-1 compared to controls (Fig. 4d and Suppl. Fig. 4B). Although SHP2 displays a strong intracellular signal, an increase of the fluorescent signal at cell boundary and/or in surface structures (e.g., membrane protrusions, membrane ruffles) was observed in PD-1 overexpressing cells as indicated by the arrows (Fig. 4d and Suppl. Fig. 4B). Furthermore, in 8505c cells transfected with PD-1-GFP, we demonstrated by IF that SHP2 and PD-1-GFP co-localize at the plasma membrane (Suppl. Fig. 4C).

### SHP2 dephosphorylates and activates Ras in TC cells

We then searched for the molecular mechanism of Ras activation mediated by the PD-1/SHP2 complex. We first asked whether PD-1 could enhance GRB2 recruitment by SHP2. To this aim, we used pull-down assays with GST-SH2-GRB2 fusion proteins and co-immunoprecipitation assays and we showed no increased GRB2 binding to SHP2 in PD-1 transfected TC cells compared to controls (Suppl. Fig. 4D). In accordance with these observations, PD-1 enforced expression did not significantly increase SHP2 tyrosine phosphorylation levels (Suppl. Fig. 4A), on which GRB2 binding to SHP2 is dependent, nor changed substantially GRB2 compartmentalization as demonstrated in cell fractionation experiments (Fig. 4c).

Since the GRB2/SOS complex is not involved in PD-1-mediated Ras activation, we asked whether SHP2 could activate Ras through the dephosphorylation of its inhibitory tyrosine residues [29, 35]. We evaluated the phosphatase activity of SHP2 and, in parallel, the levels of Ras tyrosine phosphorylation in cells overexpressing or not PD-1. We used a specific SHP2 phosphorylated substrate in the presence of the Malachite Green tracer, a colorimetric method for the detection of free inorganic phosphate [29]. We observed that SHP2 phosphatase activity was significantly increased in PD-1- versus empty-vector-transfected TC cells (Fig. 4e). Similar results were obtained in PD-1 stably transfected cells (**not shown**). Consistently with the increased phosphatase activity of SHP2, Ras total tyrosine phosphorylation levels, in the presence of PD-1, were significantly reduced in TC cells transfected with pCEFL H-Ras AU5 (Suppl. Fig. 4E). To assess whether Ras dephosphorylation occurs in its inhibitory residues 32 and/or 64 [29], we used (pan) Ras immunoprecipitation followed by immunoblotting with anti-phospho Y32 (Ras) or Y64 (Ras) antibodies. These experiments demonstrated that PD-1 enforced expression in 8505c cells reduced the Ras phosphorylation levels in the inhibitory tyrosine residues 32 in pCEFL Ras AU5-transfected cells compared to controls (Fig. 4f). Similar results were obtained in TPC-1 cells (**not shown**). No differences in phosphorylation levels of inhibitory residues 64 were observed (**not shown**).

Taken together, these data indicate that, in TC cells, PD-1 binds SHP2, which in turn dephosphorylates Ras in its inhibitory tyrosine, thus leading to the activation of the MAPK signalling cascade.

### PD-1-induced biologic activities in thyroid cancer cells require the SHP2/BRAF/MEK signalling proteins

**Fig. 5:**
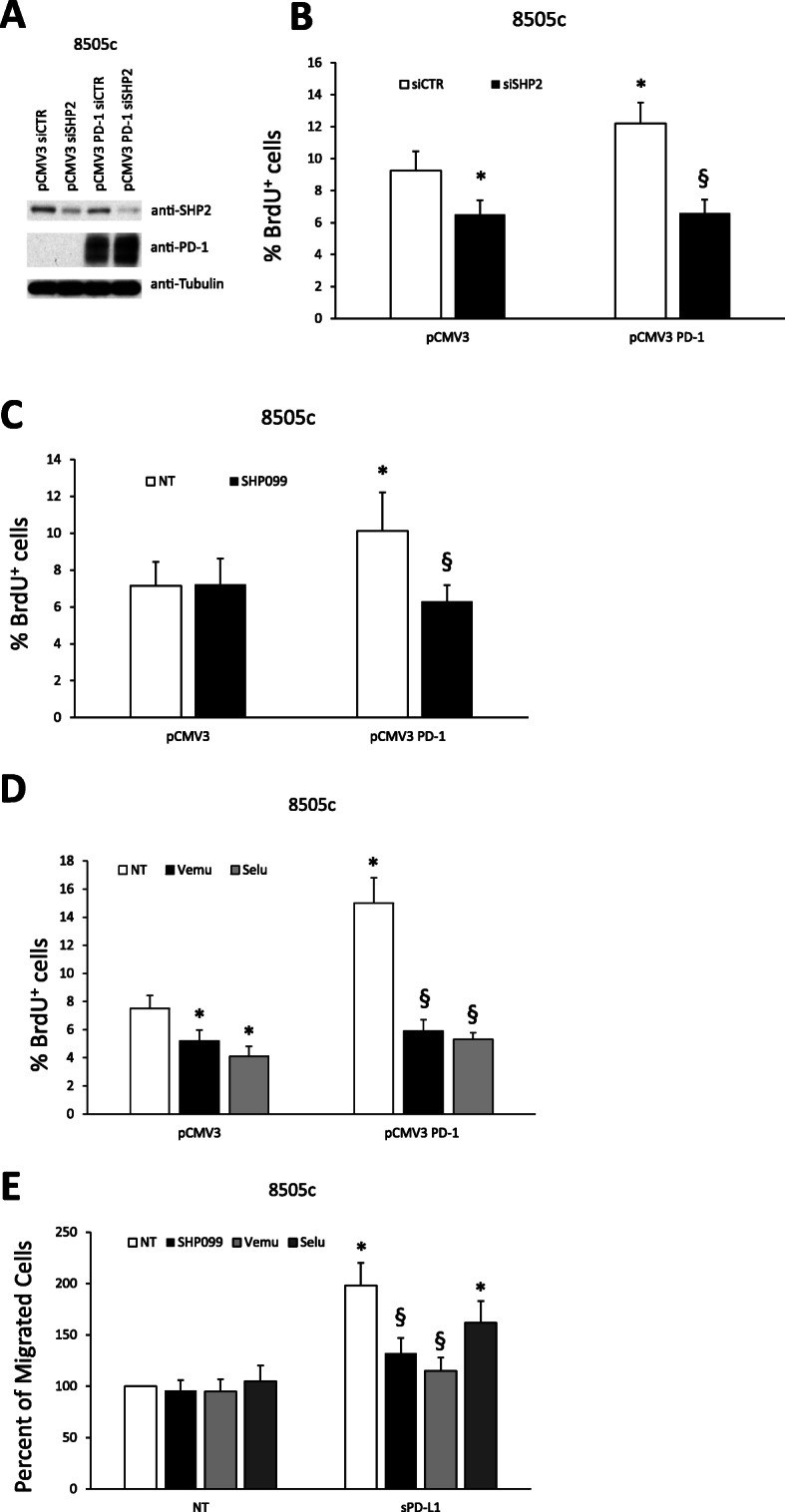
Dependence of PD-1 biologic activities on SHP2/BRAF/MEK cascade. **a.** Effects of siRNA targeting SHP2 (siSHP2–100 nM) or the relative control on SHP2 protein expression levels assessed by western blot in 8505c cells stably transfected with PD-1 or the empty vector (one representative clone is shown). **b.** DNA synthesis of stably transfected 8505c pCMV3 PD-1 cells (mean of 3 clones) compared to empty vector transfected cells treated with siSHP2 or siCTR assessed by BrdU incorporation. Data are presented as mean ± SD of 5 independent experiments. **c.** DNA synthesis of 8505c cells stably transfected with PD-1 (pCMV3 PD-1 compared to pCMV3) and treated for 18 h with SHP099 (350 nM) assessed by BrdU incorporation. The mean of 3 clones is shown for each condition. Data are presented as mean ± SD of 5 independent experiments. **d.** DNA synthesis of stably transfected 8505c pCMV3 PD-1 cells (3 clones) compared to empty vector transfected cells treated for 18 h with Vemurafenib (Vemu - 10 μM) or Selumetinib (Selu - 10 μM) assessed by BrdU incorporation. Data are presented as mean ± SD of 5 independent experiments. **e.** Percent of migrated 8505c cells over control toward sPD-L1 (1 μg/ml) or medium alone (10% FBS) following treatment with SHP099 (350 nM), Vemurafenib (Vemu - 10 μM) or Selumetinib (Selu - 10 μM). Data are presented as mean ± SD of 5 independent experiments. * *P* < 0.05 compared to the relative control. § *P* < 0.05 compared to NT or siCTR

To investigate the role of SHP2 in PD-1 functional activity, we treated TC cells, overexpressing or not PD-1, with siRNA targeting SHP2 (siSHP2–100 nM) or with a SHP2 allosteric inhibitor that blocks its phosphatase activity (SHP099–350 nM) [36]. As shown in Fig. 5a, siSHP2 was able to significantly reduce SHP2 protein levels compared to scrambled siRNAs (siCTR). By BrdU incorporation assays, we demonstrated that siSHP2 significantly decreased DNA synthesis (Fig. 5b) in PD-1-, and to a lesser extent in empty vector-transfected, 8505c cells. Consistently, SHP099 inhibitor significantly reduced PD-1-induced DNA synthesis in 8505c cells (Fig. 5c).

To investigate the role of the downstream signalling cascade in PD-1 dependent biologic TC responses, we conducted BrdU incorporation assays in TC cells overexpressing or not PD-1, in the presence or in the absence of Vemurafenib (Vemu – 10 μM) [37], a BRAF inhibitor, or Selumetinib (Selu – 10 μM) [38], a MEK inhibitor. As shown in Fig. 5d, both drugs were able to significantly revert PD-1-induced DNA synthesis in 8505c cells.

Similar experiments were performed to assess the role of the signalling cascade in PD-1-mediated TC cell migration. Figure 5e shows that SHP099 and Vemurafenib, and to a lesser extent Selumetinib, were able to inhibit the migration of 8505c cells induced by sPD-L1. Similar results were obtained in TC cells transfected with PD-1 (**not shown**).

These data demonstrate that PD-1-induced cell proliferation and motility of TC cells are dependent on the SHP2/BRAF/MEK pathway.

### Intrinsic PD-1 signalling enhances xenograft growth of TC cells in immunocompromised mice

**Fig. 6:**
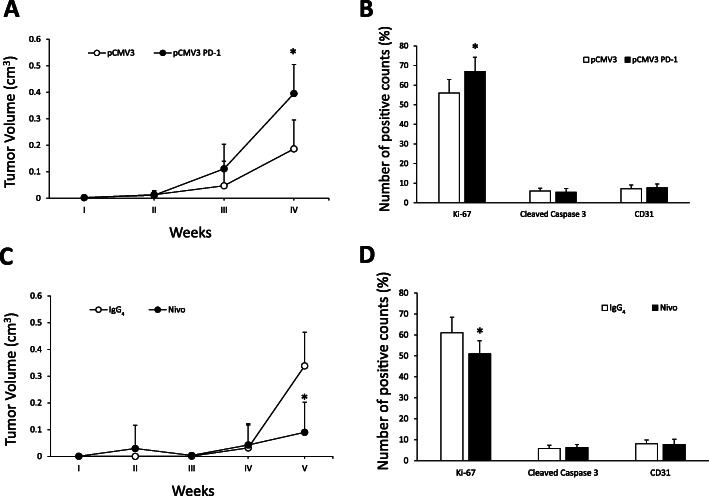
Role of intrinsic PD-1 in tumorigenicity of TC cells. **a.** Tumour growth of pCMV3- (a mass population) or PD-1-transfected (mean of 2 clones) 8505c cells. **b.** Proliferation index (Ki-67), apoptotic rate (cleaved caspase 3), and vessel density (CD31) assessed by immunohistochemistry of 8505c pCMV3 or pCMV3 PD-1 cell xenografts harvested 28 days post-inoculation. The relative quantifications (5 fields/sample) are shown. **c.** Tumour growth of 8505c xenografts in mice treated intraperitoneally (i.p.) at 30 mg/kg twice per week with Nivolumab (Nivo) or control IgG_4_. **d.** Proliferation index (Ki-67), apoptotic rate (cleaved caspase 3), and vessel density (CD31) assessed by immunohistochemistry of 8505c cell xenografts, treated with Nivoumab or IgG_4_, harvested 35 days post-inoculation. The relative quantifications (5 fields/sample) are shown.* *P* < 0.05 compared to the relative control

To verify whether PD-1 intrinsic signalling and biologic activity could affect tumorigenicity of TC cells, we xenotransplanted 8505c pCMV3 PD-1 (two clones) and control 8505c pCMV3 (a mass population) cells in athymic mice. 8505c pCMV3 PD-1 xenografts displayed increased tumour growth rate that was statistically significant at 4 weeks after injection, in comparison to empty vector transfected cells (Fig. 6a). End-stage tumours were excised and analysed for cell proliferation (Ki-67), apoptotic rate (cleaved-caspase 3) and vessel density (CD31) by immunohistochemistry. 8505c pCMV3 PD-1 and 8505c pCMV3 xenografts exhibited statistically significant differences in cell proliferation rate, but not in apoptotic rate or vessel density (Fig. 6b and Suppl. Fig. 5A).

To verify whether the inhibition of PD-1 by Nivolumab could affect xenograft growth of parental 8505c cells, mice were xenotransplanted, randomized in two homogeneous groups, and administered intraperitoneally (i.p.) with Nivolumab (Nivo) or control IgG_4_ (30 mg/kg) twice a week. 5 weeks after xenotransplantation, Nivolumab-treated tumours showed a significant decrease in growth rate in comparison with the IgG_4_-treated group (Fig. 6c). Consistently, Nivolumab significantly reduced TC xenografts’ proliferation without affecting apoptotic rate or vessel density (Fig. 6d and Suppl. Fig. 5B).

Despite these experiments were carried out in immunocompromised mice, we could not exclude that Nivolumab anti-tumour activity could be ascribed to its ability to affect innate immunity that is present and functional in athymic mice. Thus, we analysed the density and activation of immune cells infiltrating 8505c xenografts treated with Nivolumab or with IgG_4_ by cytofluorimetric analysis. We found that Nivolumab treatment did not change the percentage of CD45^+^ leucocytes infiltrating xenografts in comparison to IgG_4_ controls, at least at 5 weeks of treatment. Moreover, the density and the expression of polarization/activation markers of tumour-associated macrophages (TAM), of Ly6C^+^ and Ly6G^+^ immature myeloid cells, of mature and immature dendritic cells and of regulatory or activated NK, and NKT cells, were comparable between Nivolumab- and IgG_4_-treated 8505c xenografts (Suppl. Table 1).

These data indicate that, in our model system, PD-1 blockade by Nivolumab inhibits TC cell xenograft growth by affecting tumour cell rather than immune cell compartment.

## Discussion

Several reports point to a promising role of immunotherapy in the treatment of advanced forms of TCs [15, 39]. TCGA analysis of TC provided a classification of PTC, in spite of their low mutational burden, as “inflamed” tumours and ATC as hot tumours [40]. Interestingly, a profiling of TC confirmed that ATC and PTC are strongly infiltrated by macrophages and CD8^+^ T cells, but that these cells displayed a functionally exhausted appearance [11]. In TC, high PD-L1 levels significantly correlated with immune infiltrate, increased tumour size and multifocality [17, 18]. Furthermore, the presence of PD-1^+^ T lymphocyte infiltrating TC is associated with lymph-nodal metastasis and recurrence [13]. Altogether, these data suggest that immune checkpoint inhibitors (ICI) might represent a promising tool for the treatment of these carcinomas.

Our report, for the first time, investigated the expression of the PD-1 receptor in epithelial thyroid cancer cells, demonstrating that a significant percentage of human TC samples displayed PD-1 expression on these cells, although at lower levels compared to the expression found on immune cells infiltrating the tumour. Consistently with the evidence obtained for PD-L1 [17, 41], our data indicate that PD-1 expression levels correlated with tumour stage and lymph-nodal metastasis in TC. Accordingly, we demonstrated that PD-1 activity could induce proliferation and motility of TC cells in culture. This suggests that the PD-1 intrinsic pathway might have a role in TC cell aggressiveness and invasive ability.

The expression of PD-1 on cancer cells, rather than on immune cells, has been observed recently in melanoma and hepatocellular carcinoma (HCC) [5, 6, 42] where PD-1 exerts a tumor promoting function. On the other hand, the intrinsic PD-1 expression in non-small cell lung cancer (NSCLC) has been described to exert a tumor suppressor role and to represent a potential mechanism by which PD-1 blockade may promote cancer growth [7, 42].

In melanoma and HCC, intrinsic PD-1 activity sustains tumour growth through an mTOR/S6K1 signalling [5, 6, 42]. In TC cells, similarly to melanoma and HCC, PD-1 intrinsic signalling sustains cancer cell proliferation, but at variance from these neoplasias, this biologic activity is mediated by the activation of the Ras/MAPK pathway. Interestingly, mutations causing the activation of the Ras/MAPK signalling pathway are found in > 70% of PTC (e.g., *RET/PTC* rearrangements and point mutations of the *BRAF* and *Ras* genes) and regulate transcription of key genes involved in TC cell proliferation [43]. PD-1 expression could provide a selective advantage to some TC by enhancing the activation of MAPK pathway, thus promoting proliferation and migratory behaviour of cancer cells. Interestingly, besides PD-1, also the immune-checkpoint Cytotoxic T lymphocyte-associated antigen 4 (CTLA-4), classically expressed on leukocytes, has been found to be expressed and functional on cancer cells [44, 45].

Our data also highlighted the key role of the SHP2 tyrosine-phosphatase in PD-1-mediated activities in TC cells. Interestingly, SHP2 is recruited by PD-1 in T lymphocytes, and inhibits immune receptor signalling by dephosphorylating several downstream substrates [31, 46]. In cancer cells, SHP2 has been described to exhibit oncogenic properties [32, 33]. SHP2 functions as an adapter that binds activated receptor tyrosine kinases (RTKs) and recruits the GRB2/SOS complex on the plasma membrane, enhancing SOS-mediated GTP loading on Ras and activating the Ras/MAPK cascade [32, 33]. SHP2 can also directly enhance Ras activity by dephosphorylating specific inhibitory tyrosine residues on Ras [29, 35, 47]. In our model system, we found that PD-1 exploits this last mechanism. However, we cannot exclude that other PD-1 functions may contribute to Ras/MAPK activation. Whatever the case, we demonstrated that, in TC cells, SHP2 is a critical factor in PD-1 downstream signalling, as SHP2 inhibition hampered PD-1-mediated biological activities.

The majority of TC are driven by mutations that activate the Ras/MAPK pathway. Inhibitors targeting different proteins in this signalling cascade have been developed, but their efficacy has been limited by adaptive feedback reactivation of the pathway [48]. Interestingly, SHP2 has been identified as one of the main mediators of adaptive resistance to inhibitors of the Ras/MAPK pathway in many tumors, including TC. In 8505c cells, carrying a BRAF(V600E) mutation, targeting both BRAF and SHP2 with Vemurafenib and SHP099 led to a reversion of adaptive resistance to either inhibitor alone [49, 50]. Furthermore, in TC samples, increased SHP2 expression was detected compared to normal thyroids, and this correlated with poor tumour differentiation, TNM stage and lymph-nodal metastasis [51]. These evidences suggest that SHP2 may represent a potential target for TC therapy both alone and in combination with PD-1 and/or Ras/MAPK targeting.

The evaluation of PD-1 expression in cancer cell might be important to identify tumours and/or patients that will be likely to respond to ICI administration by taking advantage of both drug effects on immune compartment and on cancer cell proliferation. In few case reports or in “basket clinical trials” in which ICI [i.e., Pembrolizumab (anti-PD-1), Nivolumab (anti-PD-1), or Atezolizumab (anti-PD-L1)] were used alone or in combination with Multikinase Inhibitors (MKI) for the treatment of advanced and/or metastatic TC, encouraging preliminary clinic evidence of efficacy has been reported [9, 52, 53].

## Conclusions

Our observations demonstrate that PD-1 is expressed on TC cells and exerts an intrinsic pro-tumorigenic function. Thus TC, and possibly other cancer types, could benefit of the dual effects of ICI: the reactivation of immune anti-tumour response and the direct anti-proliferative effects on cancer cells. Defining the functional and biochemical activity of immune checkpoints both in cancerous cells and in tumour microenvironment of TC will expand our knowledge allowing to develop rational therapeutic strategies for aggressive TC exploiting ICI in combination with SHP2 or RTK/Ras/MAPK inhibitors.

## Supplementary Information

**Additional file 1: Supplementary Table 1.** Mouse immune cell density (expressed as percentage of CD45^+^ leukocytes) in 8505c xenografts.

**Additional file 2: Supplementary Figure 1.** Immune checkpoint expression in thyroid cancer (TC) cells. Protein expression levels assessed by western blot **(A)** and mRNA expression indicated as ΔCt for PD-1, PD-L1 and PD-L2 **(B)** in H-6040 normal thyroid epithelial cells, PTC-derived cell lines (BcPAP and TPC-1), and ATC-derived cell lines (8505c, CAL62, SW1736, FRO, BHT101, HTH7, OCUT1). A representative western blot experiment is shown. PCR data are presented as mean ± SD of 5 independent experiments.

**Additional file 3: Supplementary Figure 2.** Functional activity of intrinsic PD-1 circuit in TC cells. **A.** Expression levels of PD-1 in 8505c and TPC1 cells or in 8505c and TPC-1 transiently transfected with pFLAG or pFLAG PD-1, assessed by western blot. A representative experiment is shown. **B.** Cell cycle distribution of 8505c and TPC-1 cells transiently transfected with pFLAG or pFLAG PD-1, measured by Propidium Iodide (PI) staining by means of Flow Cytometry. The percent of the cells distributed in G0/G1, S, G2/M was indicated in each panel. Representative experiments are shown. **C.** Percent of apoptotic cells assessed by TUNEL reaction in 8505c and TPC-1 cells transiently transfected with pFLAG or pFLAG PD-1 and treated or not with soluble PD-L1 (sPD-L1 - 1 μg/ml). Data are presented as mean ± SD of 5 independent experiments. **D.** Cytofluorimetric evaluation of PD-1 expression in 8505c cells treated with siPD-1 (solid lines) or scrambled siCTR (dotted line) (100 nM). A representative experiment is shown. **E.** Cell cycle distribution of 8505c and TPC-1 cells treated with Nivolumab (Nivo - 10 μg/ml) or control IgG_4_ (10 μg/ml), measured by Propidium Iodide (PI) staining by means of Flow Cytometry. The percent of the cells distributed in G0/G1, S, G2/M was indicated in each panel. Representative experiments are shown. **F.** Percent of apoptotic cells assessed by TUNEL reaction in 8505c and TPC-1 cells treated with siPD-1 (100 nM) or Nivolumab (Nivo - 10 μg/ml) or the relative controls. Data are presented as mean ± SD of 5 independent experiments. **G.** DNA synthesis of 8505c cells transiently transfected with pCMV3, pCMV3 PD-L1 or pCMV3 PD-L2 or treated with anti-PD-L1, anti-PD-L2 blocking antibodies or IgG_1_ isotype control (10 μg/ml) assessed by BrdU incorporation. Data are presented as mean ± SD of 5 independent experiments. * *P*<0.05 compared to the relative control.

**Additional file 4: Supplementary Figure 3.** Signalling pathways downstream PD-1overexpression. **A.** Expression levels of PD-1 in some clones or mass populations obtained from 8505c cells stably transfection with PD-1, assessed by western blot. A representative experiment is shown. **B**. Expression levels of phosphorylated forms of BRAF, MEK1/2 and MAPK (p44/p42) in 8505c cells stably transfected with PD-1 or the empty vector, assessed by western blot. A representative experiment is shown. **C**. Activation of AKT, SRC, S6, S6K, 4EBP1 in 8505c and TPC-1 cells, transiently transfected or not with PD-1 or the relative empty vector, assessed by western blot for their phosphorylated forms. A representative experiment is shown.

**Additional file 5: Supplementary Figure 4.** Effects of intrinsic PD-1 on SHP2 localization and functions. **A.** Expression levels of PD-1, phospho-PD-1, SHP2 and phospho-SHP2 in 8505c and TPC-1 cells transiently transfected with pFLAG PD-1 or the empty vector pFLAG, assessed by western blot. A representative experiment is shown. **B.** Immunofluorescence microscopy of 8505c cells, transiently transfected with pFLAG PD-1 or the empty vector, with antibody specific for SHP2. Arrows indicate the surface signal of SHP2. Bars, 5 μm. A representative experiment is shown. **C.** Immunofluorescence microscopy of 8505c cells transiently transfected with pCMV6 PD-1-GFP and stained with antibody specific for SHP2, and the merged signal. Arrows indicate the surface signal of SHP2. Bars, 5 μm. A representative experiment is shown. **D.** Total protein extracts from TPC-1 cells transiently transfected with pFLAG-PD-1 or the empty vector pFLAG were subjected to a pull-down assay using the indicated recombinant proteins or to immunoprecipitation using the indicated antibodies. Proteins were immunoblotted with antibody against SHP2 or GRB2. A representative experiment is shown. **E**. Total cell protein extracts from 8505c cells transiently transfected with combination of pCEFL H-Ras AU5, pFLAG PD-1 or empty vector (pFLAG + pCEFL) were subjected to immunoprecipitation with anti-phospho tyrosine followed by western blotting with pan (RAS) antibody. A representative experiment is shown, together with the mean densitometric analysis ± SD of 5 independent assays. * P<0.05 compared to the relative control.

**Additional file 6: Supplementary Figure 5.** Immunohistochemical evaluation of 8505c xenografts. A. Proliferation index (Ki-67) assessed by immunohistochemistry of 8505c pCMV3 and pCMV3 PD-1 cl13 xenografts harvested 28 days post-inoculation. Representative images are shown. **B.** Proliferation index (Ki-67) assessed by immunohistochemistry of 8505c xenografts harvested 35 days post-inoculation in mice treated with Nivolumab or control IgG_4_. Representative images are shown.

## Data Availability

All data generated or analysed during this study are included within the article or available from the corresponding author on reasonable request.

## Abbreviations

PD-L1: Programmed cell death-ligand 1
PD-L2: Programmed cell death-ligand 2
IC: Immune checkpoints
PD-1: Programmed cell death-1
ICI: Immune checkpoint inhibitors
moAb: Monoclonal antibody
mTOR: Mammalian target of rapamycin
S6K1: Ribosomal protein S6 kinase
NSCLS: Non-small cell lung cancer
TC: Thyroid carcinoma
WDTC: Well differentiated thyroid carcinoma
PDTC: Poorly differentiated thyroid carcinoma
ATC: Anaplastic thyroid carcinoma
PTC: Papillary thyroid carcinoma
FTC: Follicular thyroid carcinoma
RAI: Radioactive iodine I-131
SHP-2: Src homology 2 (SH2)-containing protein tyrosine phosphatase 2
RAS: Rat sarcoma
MAPK: Mitogen-activated protein kinases
FBS: Fetal bovine serum
BRAF: Serine/threonine-protein kinase B-raf
MEK1/2: Mitogen-activated protein kinase kinase 1
GRB2: Growth factor receptor bound protein 2
GST: Glutathione S-transferase
SDS-PAGE: Sulphate-polyacrylamide gel electrophoresis
ECL: Enhanced chemiluminescence
PBS: Phosphate buffered saline
PFA: Paraformaldehyde
BSA: Bovine serum albumin
IgG_4_: Immunoglobulin G_4_
Ki-67: Marker of proliferation Ki-67
CD31: Cluster of differentiation 31
SEM: Standard error of the mean
IHC: Immunohistochemistry
sPD-L1: Soluble PD-L1
siRNA: Small interfering RNA
BrdU: Bromodeoxyuridine
Src: Proto-oncogene tyrosine-protein kinase Src
S6: Ribosomal protein S6
AKT: Protein kinase B
4EBP1: Eukaryotic translation initiation factor 4E-binding protein 1
RBD: Ras binding domain
PTPN11: Tyrosine-protein phosphatase non-receptor type 11
SOS: Son of sevenless
GTP: Guanosine triphosphate
TAM: Tumour associated macrophages
Ly6C^+^: Lymphocyte antigen 6C
Ly6G^+^: Lymphocyte antigen 6G
NK: Natural killer
NKT: Natural killer T
HCC: Hepatocellular carcinoma
CTLA-4: Cytotoxic T lymphocyte-associated antigen 4
RET: Proto-oncogene tyrosine-protein kinase receptor Ret
TNM: Tumour-nodes-metastasis
MKI: Multi-kinase inhibitors
