# Supplementary figures and images for: PD-1 blockade delays tumor growth by inhibiting an intrinsic SHP2/Ras/MAPK signalling in thyroid cancer cells
Source: J Exp Clin Cancer Res. 2021 Jan 7;40:22. doi: 10.1186/s13046-020-01818-1 (PMC7791757; doi:10.1186/s13046-020-01818-1)

**A**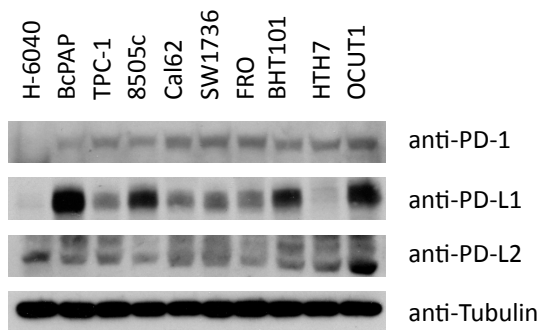**B**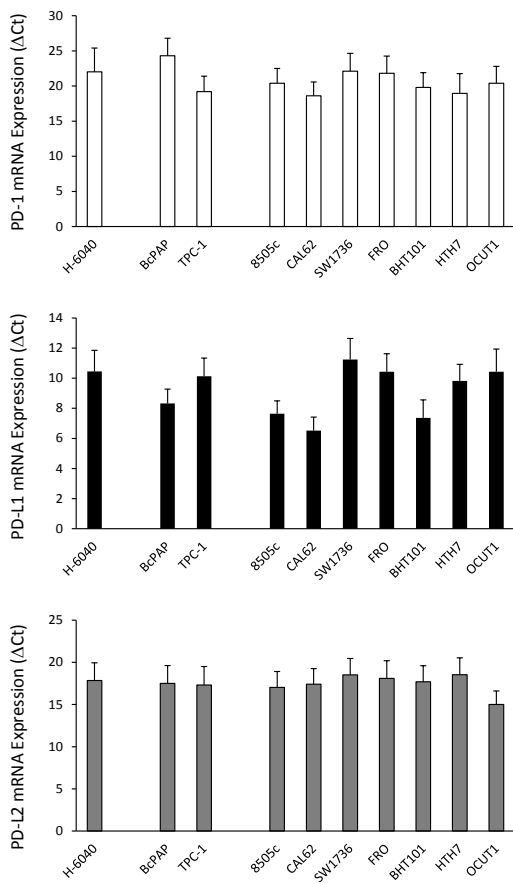

Supplement: Supplementary file 2 — Additional file 2: Supplementary Figure 1. Immune checkpoint expression in thyroid cancer (TC) cells. Protein expression levels assessed by western blot (A) and mRNA expression indicated as ΔCt for PD-1, PD-L1 and PD-L2 (B) in H-6040 normal thyroid epithelial cells, PTC-derived cell lines (BcPAP and TPC-1), and ATC-derived cell lines (8505c, CAL62, SW1736, FRO, BHT101, HTH7, OCUT1). A representative western blot experiment is shown. PCR data are presented as mean ± SD of 5 independent experiments. [file 13046_2020_1818_MOESM2_ESM.pdf]

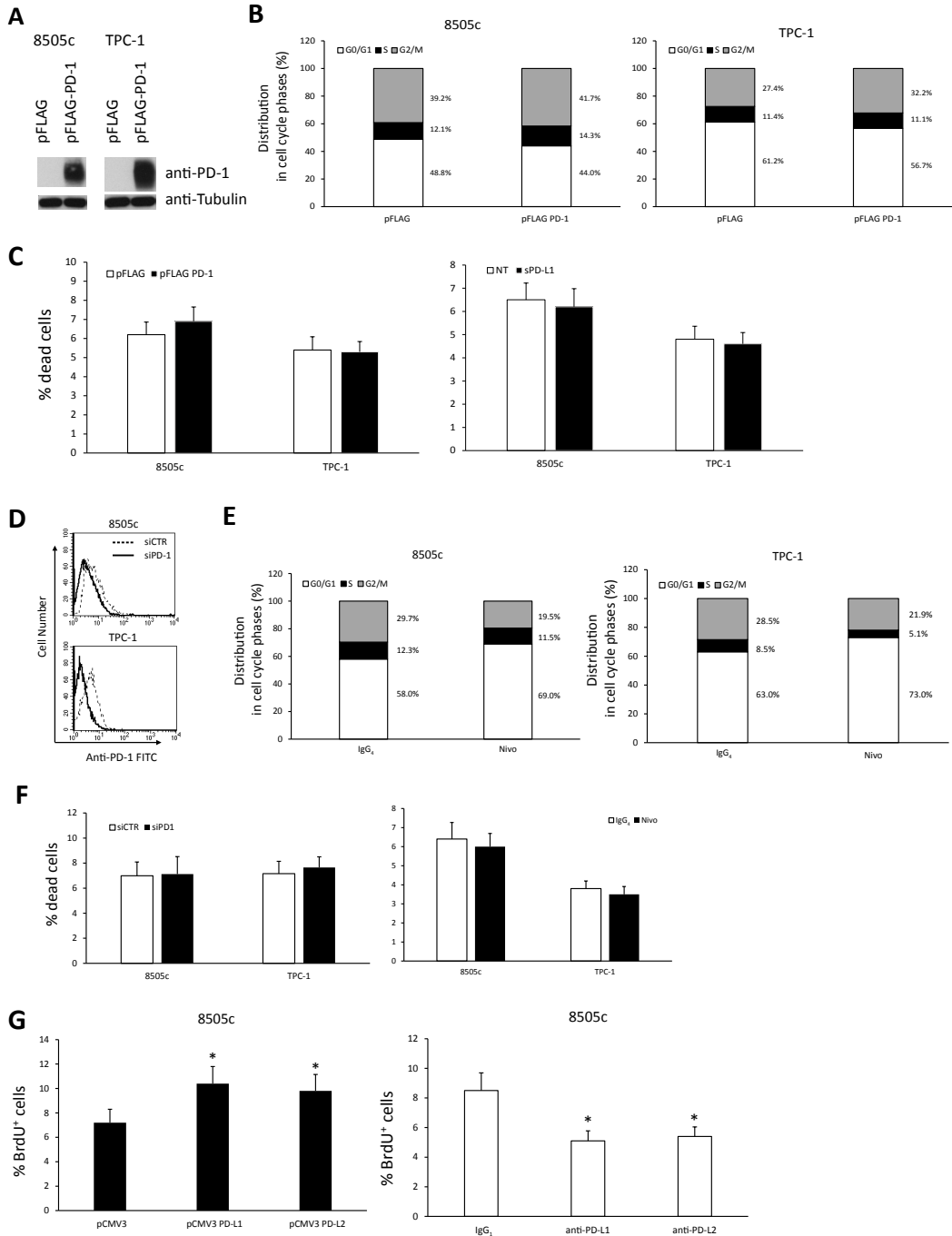

Supplement: Supplementary file 3 — Additional file 3: Supplementary Figure 2. Functional activity of intrinsic PD-1 circuit in TC cells. A. Expression levels of PD-1 in 8505c and TPC1 cells or in 8505c and TPC-1 transiently transfected with pFLAG or pFLAG PD-1, assessed by western blot. A representative experiment is shown. B. Cell cycle distribution of 8505c and TPC-1 cells transiently transfected with pFLAG or pFLAG PD-1, measured by Propidium Iodide (PI) staining by means of Flow Cytometry. The percent of the cells distributed in G0/G1, S, G2/M was indicated in each panel. Representative experiments are shown. C. Percent of apoptotic cells assessed by TUNEL reaction in 8505c and TPC-1 cells transiently transfected with pFLAG or pFLAG PD-1 and treated or not with soluble PD-L1 (sPD-L1 - 1 μg/ml). Data are presented as mean ± SD of 5 independent experiments. D. Cytofluorimetric evaluation of PD-1 expression in 8505c cells treated with siPD-1 (solid lines) or scrambled siCTR (dotted line) (100 nM). A representative experiment is shown. E. Cell cycle distribution of 8505c and TPC-1 cells treated with Nivolumab (Nivo - 10 μg/ml) or control IgG4 (10 μg/ml), measured by Propidium Iodide (PI) staining by means of Flow Cytometry. The percent of the cells distributed in G0/G1, S, G2/M was indicated in each panel. Representative experiments are shown. F. Percent of apoptotic cells assessed by TUNEL reaction in 8505c and TPC-1 cells treated with siPD-1 (100 nM) or Nivolumab (Nivo - 10 μg/ml) or the relative controls. Data are presented as mean ± SD of 5 independent experiments. G. DNA synthesis of 8505c cells transiently transfected with pCMV3, pCMV3 PD-L1 or pCMV3 PD-L2 or treated with anti-PD-L1, anti-PD-L2 blocking antibodies or IgG1 isotype control (10 μg/ml) assessed by BrdU incorporation. Data are presented as mean ± SD of 5 independent experiments. * P<0.05 compared to the relative control. [file 13046_2020_1818_MOESM3_ESM.pdf]

8505c

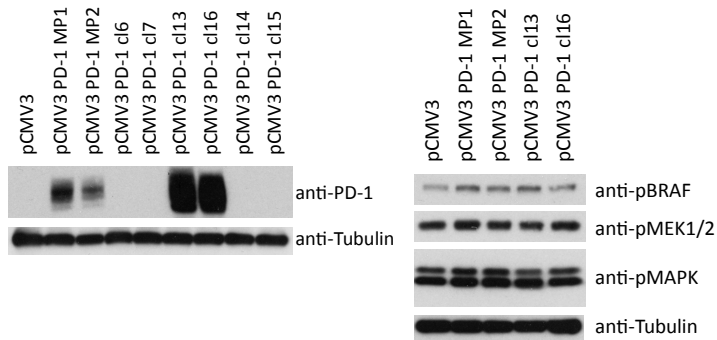

**C**

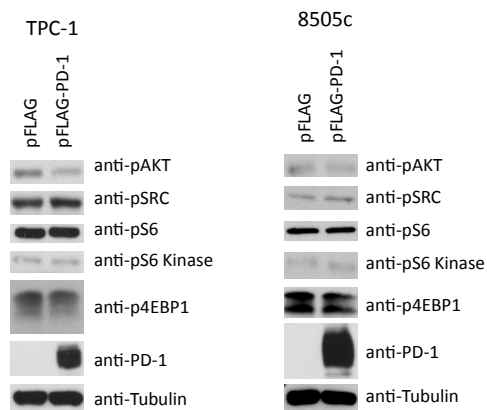

Supplement: Supplementary file 4 — Additional file 4: Supplementary Figure 3. Signalling pathways downstream PD-1overexpression. A. Expression levels of PD-1 in some clones or mass populations obtained from 8505c cells stably transfection with PD-1, assessed by western blot. A representative experiment is shown. B. Expression levels of phosphorylated forms of BRAF, MEK1/2 and MAPK (p44/p42) in 8505c cells stably transfected with PD-1 or the empty vector, assessed by western blot. A representative experiment is shown. C. Activation of AKT, SRC, S6, S6K, 4EBP1 in 8505c and TPC-1 cells, transiently transfected or not with PD-1 or the relative empty vector, assessed by western blot for their phosphorylated forms. A representative experiment is shown. [file 13046_2020_1818_MOESM4_ESM.pdf]

**A**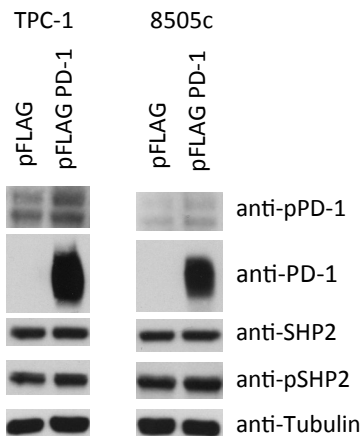**B**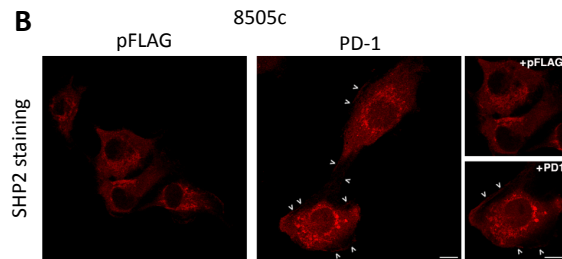**C**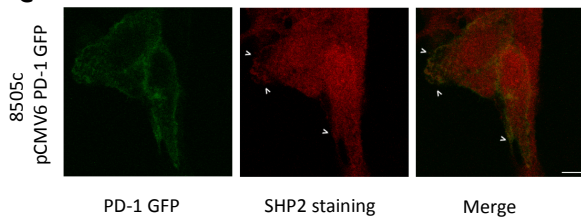**D**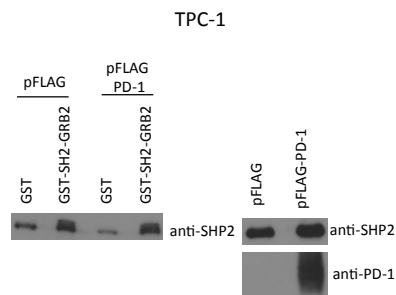

TPC-1

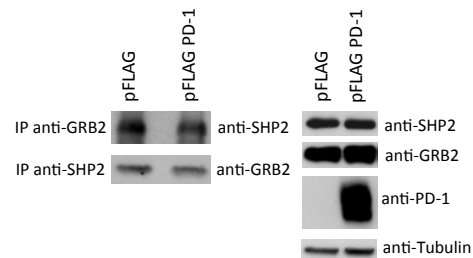**E**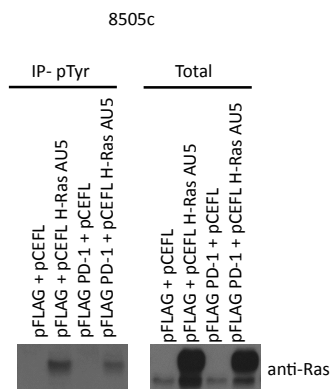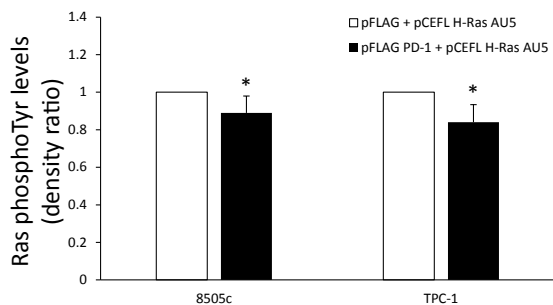

Supplement: Supplementary file 5 — Additional file 5: Supplementary Figure 4. Effects of intrinsic PD-1 on SHP2 localization and functions. A. Expression levels of PD-1, phospho-PD-1, SHP2 and phospho-SHP2 in 8505c and TPC-1 cells transiently transfected with pFLAG PD-1 or the empty vector pFLAG, assessed by western blot. A representative experiment is shown. B. Immunofluorescence microscopy of 8505c cells, transiently transfected with pFLAG PD-1 or the empty vector, with antibody specific for SHP2. Arrows indicate the surface signal of SHP2. Bars, 5 μm. A representative experiment is shown. C. Immunofluorescence microscopy of 8505c cells transiently transfected with pCMV6 PD-1-GFP and stained with antibody specific for SHP2, and the merged signal. Arrows indicate the surface signal of SHP2. Bars, 5 μm. A representative experiment is shown. D. Total protein extracts from TPC-1 cells transiently transfected with pFLAG-PD-1 or the empty vector pFLAG were subjected to a pull-down assay using the indicated recombinant proteins or to immunoprecipitation using the indicated antibodies. Proteins were immunoblotted with antibody against SHP2 or GRB2. A representative experiment is shown. E. Total cell protein extracts from 8505c cells transiently transfected with combination of pCEFL H-Ras AU5, pFLAG PD-1 or empty vector (pFLAG + pCEFL) were subjected to immunoprecipitation with anti-phospho tyrosine followed by western blotting with pan (RAS) antibody. A representative experiment is shown, together with the mean densitometric analysis ± SD of 5 independent assays. * P<0.05 compared to the relative control. [file 13046_2020_1818_MOESM5_ESM.pdf]

**A**

8505c

pCMV3

pCMV3 PD-1 CI 13

Ki-67 staining

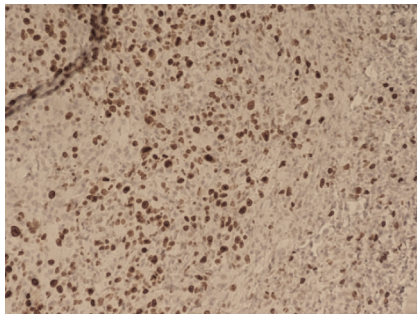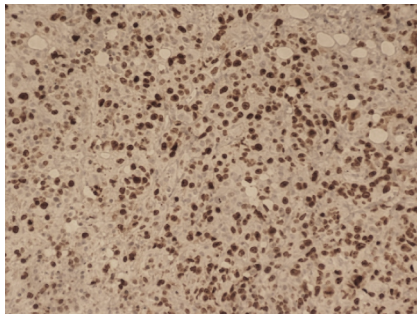**B**

8505c

Ki-67 staining

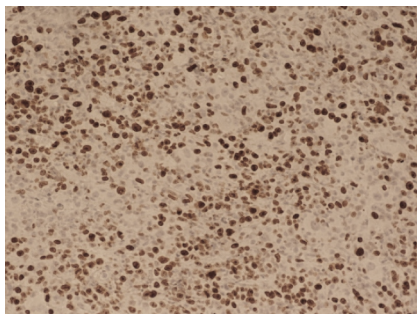IgG<sub>4</sub>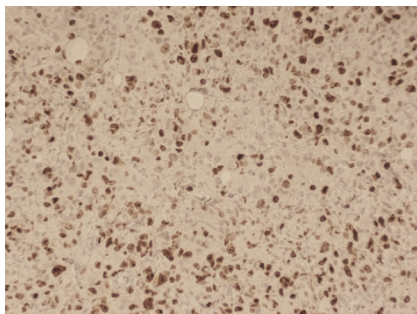

Nivolumab

Supplement: Supplementary file 6 — Additional file 6: Supplementary Figure 5. Immunohistochemical evaluation of 8505c xenografts. A. Proliferation index (Ki-67) assessed by immunohistochemistry of 8505c pCMV3 and pCMV3 PD-1 cl13 xenografts harvested 28 days post-inoculation. Representative images are shown. B. Proliferation index (Ki-67) assessed by immunohistochemistry of 8505c xenografts harvested 35 days post-inoculation in mice treated with Nivolumab or control IgG4. Representative images are shown. [file 13046_2020_1818_MOESM6_ESM.pdf]
